# Organising Joint Primary & Secondary Care (CPA) Reviews for severe mental health disorders hosted at GP practice in Walsall UK, an exemplar of collaborative working

**DOI:** 10.1192/j.eurpsy.2022.1569

**Published:** 2022-09-01

**Authors:** S.S. Afghan, A. Shanker

**Affiliations:** 1 Dorothy Pattison Hospital, Adult Mental Health, Walsall, United Kingdom; 2 New Invention Health Centre, Walsall Clinical Commissioning Group, Walsall, United Kingdom

**Keywords:** Service innovation, primary & secondary care collaboration, Multidisciplinary,

## Abstract

**Introduction:**

Provision of holisitc, accessible and high quality mental health care to the patients requires sharing of responsibilities & resources, enhanced communication & collaboration at the interface (primary care systems and secondary care mental health services). An innovative model of single point of care for people with severe and enduring mental health problems hosted at a primary care (GP) setting has been developed and evaluated in Walsall, UK.

**Objectives:**

To develop and evaluate an integrated (multidisciplinary) approach of managing health & social care needs of people with severe mental health disorders.

**Methods:**

People with severe & enduring mental health problems were reviewed in primary care (N=65). A comprehensive physical, mental and psychosocial assessments were undertaken by the clinicians that included GP, Psychiatrist and Care-Coordinator.The reviews included: 1) A review of physical health indicators based on the Lester toolkit by practice pharmacist/nurse, including lifestyle, body weight, BMI and blood pressure. 2) Individualised interventions included physical / psychiatric prescribing, social prescribing and advise on lifestyle changes. Stable patients were recommended for stepping down from the secondary care. Outcomes included Patient Satisfaction Questionnaire (PSQ).

**Results:**

Satisfaction on the PSQ was rated from very good to excellent. Results highlighted multiple benefits including trust generation, improved communication among professionals, physical health screening and prompt clinical decision making (e.g. referral / prescribing). Other benefits included patient access & satisfaction, time and cost efficiency by reducing the number of reviews.

**Conclusions:**

The integtrated CPA reviews offers efficient, holisitc & cost effective model of care with high satisfaction levels for patients and is replicable.

**Disclosure:**

No significant relationships.

